# Cerebral trauma-induced dyschromatopsia in the left hemifield: case presentation

**DOI:** 10.1186/s12886-020-01800-7

**Published:** 2021-01-27

**Authors:** Yoko Mase, Yoshitsugu Matsui, Eriko Uchiyama, Hisashi Matsubara, Masahiko Sugimoto, Akiko Kubo, Mineo Kondo

**Affiliations:** 1 Department of Ophthalmology, Mie University Graduate School of Medicine, 2-174 Edobashi, Tsu City, Mie 514-8507 Japan; 2 Kinan Hospital, 4750 Atawa, Mihama Town, Mie 519-5204 Japan; 3 The Jikei University Hospital, 3-19-18 Nishishinnbashi, Minato-ku, Tokyo, 105-0003 Japan

**Keywords:** Acquired cerebral color anomaly, Cerebral dyschromatopsia, Hemilateral dyschromatopsia, SWAP, GCC thinning, Retrograde transsynaptic degeneration, Fusiform gyrus, Lingual gyrus

## Abstract

**Background:**

Acquired color anomalies caused by cerebral trauma are classified as either achromatopsias or dyschromatopsias (Zeki, Brain 113:1721–1777, 1990). The three main brain regions stimulated by color are V1, the lingual gyrus, which was designated as human V4 (hV4), and the fusiform gyrus, designated as V4α. (Zeki, Brain 113:1721–1777, 1990). An acquired cerebral color anomaly is often accompanied by visual field loss (hemi- and quadrantanopia), facial agnosia, prosopagnosia, visual agnosia, and anosognosia depending on the underlying pathology (Bartels and Zeki, Eur J Neurosci 12:172–193, 2000), (Meadows, Brain 97:615–632, 1974), (Pearman et al., Ann Neurol 5:253–261, 1979). The purpose of this study was to determine the characteristics of a patient who developed dyschromatopsia following a traumatic injury to her brain.

**Case presentation:**

The patient was a 24-year-old woman who had a contusion to her right anterior temporal lobe. After the injury, she noticed color distortion and that blue objects appeared green in the left half of the visual field. Although conventional color vision tests did not detect any color vision abnormalities, short wavelength automated perimetry (SWAP) showed a decrease in sensitivity consistent with a left hemi-dyschromatopsia. Magnetic resonance imaging (MRI) detected abnormalities in the right fusiform gyrus, a part of the anterior temporal lobe. At follow-up 14 months later, subjective symptoms had disappeared, but the SWAP abnormalities persisted and a thinning of the sectorial ganglion cell complex (GCC) was detected.

**Conclusion:**

The results indicate that although the subjective symptoms resolved early, a reduced sensitivity of SWAP remained and the optical coherence tomography (OCT) showed GCC thinning. We conclude that local abnormalities in the anterior section of fusiform gyrus can cause mild cerebral dyschromatopsia without other symptoms. These findings indicate that it is important to listen to the symptoms of the patient and perform appropriate tests including the SWAP and OCT at the early stage to objectively prove the presence of acquired cerebral color anomaly.

## Background

Acquired color anomalies caused by cerebral trauma are classified as achromatopsias or dyschromatopsias. In achromatopsia there is complete absence of color perception and patients complain that everything appears in shades of gray. In dyschromatopsia the discrimination of the main hues is still preserved, but the ability to distinguish between fine shades of color is reduced. Achromatopsia is caused by bilateral lesions of the lingual and fusiform gyri, while hemiachromatopsia occurs with unilateral right- or left-sided lesions. It has been shown that brain damage in the visual cortex and the V4 lead to achromatopsia, however the extent of the lesions cannot be determined solely from patent symptoms [1].

Color anomalies caused by alterations of the visual cortex are often accompanied by other symptoms such as prosopagnosia, an inability to recognize the faces of familiar people, and visual field defects [2–4]. In addition, Nakadomari and colleagues reported that 88% of 83 cases of cerebral dyschromatopsia had visual field defects with complications. They also reported that 72% had prosopagnosia, 43% had geographic disorientation, 20% had dyslexia, and 16% had visual agnosia [5].

Some acquired color vision defects with accompanying anosognosia has been reported [6–8]. Malihi and colleagues reported on a patient with a head trauma on the right side who had neither an awareness of color vision abnormalities nor the presence of MRI abnormalities. But when the patient was examined as a glaucoma suspect 50 years after the trauma, short wavelength automated perimetry (SWAP) detected a reduction in sensitivity to short wavelength light even though the standard automated perimetry (SAP) findings were normal [8].

We present our findings in a case of dyschromatopsia that developed after a head trauma.

## Case presentation

We hereby present the case of a 24-year-old woman with trauma-induced cerebral dyschromatopsia. The patient had a brain contusion on the right anterior temporal lobe, a part of the fusiform gyrus due to automobile accident. When she woke up after the trauma, she thought the right half of the hospital staffs’ uniform was blue and the left half was green, and she thought “it was very fashionable”. But when she told her family about it, she realized that the uniform was actually all blue, and 3 days after the injury she visited our department.

**Fig. 1:**
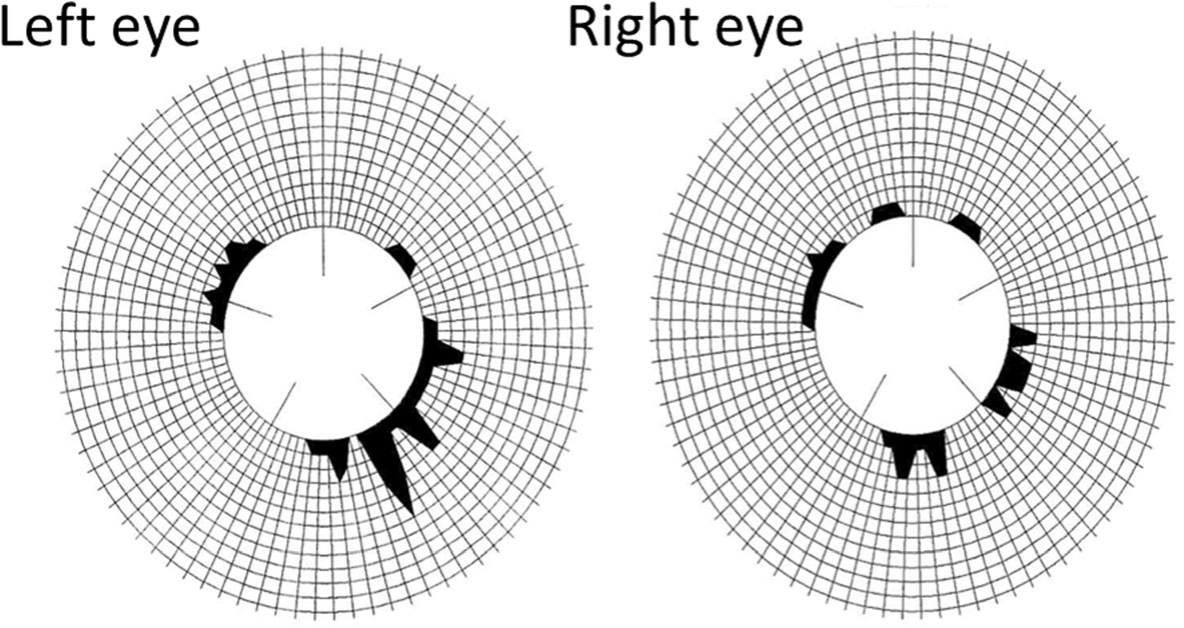
Results of Farnsworth-Munsell 100-hue test with central fixation of a patient following traumatic injury of the right side of the head. The patient was diagnosed with cerebral dyschromatopsia. In right eye, the total Error Score was 52, the orientation axis was 10.69, and the degree of polarity was 0.68. In left eye, the total Error Score was 68, the orientation axis was 12.25, and the degree of polarity was 1.13. The results of both eyes were within normal limits

**Fig. 2:**
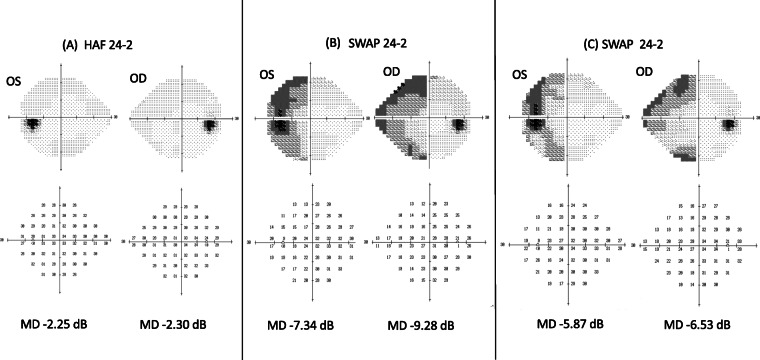
Visual field tests in same patient with cerebral dyschromatopsia. **a** Standard automated perimetry (SAP) 3 days after injury. The fields are essentially normal within the 24 degrees field. **b** Short-wavelength automated perimetry (SWAP) at 1 month after the injury. The sensitivity in the left half of the 24 degree visual field is reduced. **c** Short-wavelength automated perimetry (SWAP) at 22 months after the injury. The sensitivity in the left half of the 24 degree visual field is slightly improved

**Fig. 3:**
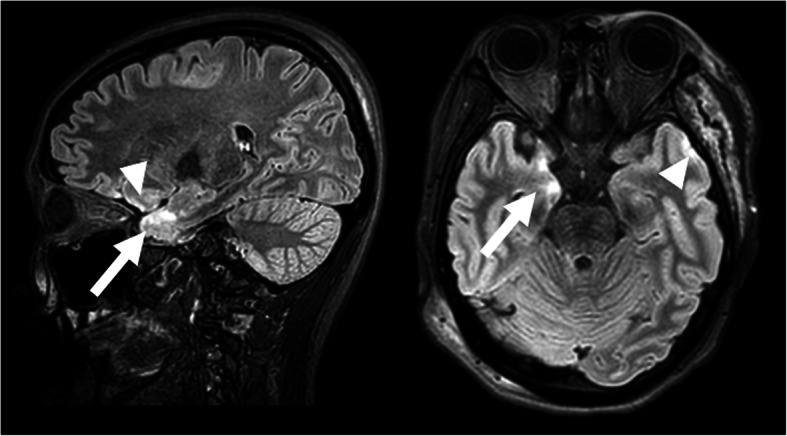
Fluid attenuated inversion recovery (FLAIR) imaging of head magnetic resonance imaging (MRI) in the same patient with cerebral dyschromatopsia. Right figure is a sagittal image, and left figure is an axial image at 3 days after injury. There were signs of brain contusion in the right anterior temporal lobe (arrows) and the presence of subarachnoid hemorrhages (arrowheads)

At the initial examination, her visual acuity was 20/16 OU, and the intraocular pressure was 18 mmHg OU. There were no significant findings in the anterior segment of the eye, media, or fundus. She passed the Panel D-15 hue arrangement test with one error, and the results of the Farnsworth-Munsell 100-hue test were within the normal limits (Fig. 1). Full-field electroretinography (ERG) was within the normal range including the S-cone ERGs. Neither Goldmann perimetry nor standard automated perimetry (SAP) showed any abnormal findings (Fig. 2a), but SWAP showed a decrease in sensitivity in the left hemilateral fields (Fig. 2b). Magnetic resonance imaging (MRI) of the brain revealed evidence of the cerebral contusions on the right anterior temporal lobe and subarachnoid hemorrhages in the base of brain (Fig. 3).

**Fig. 4:**
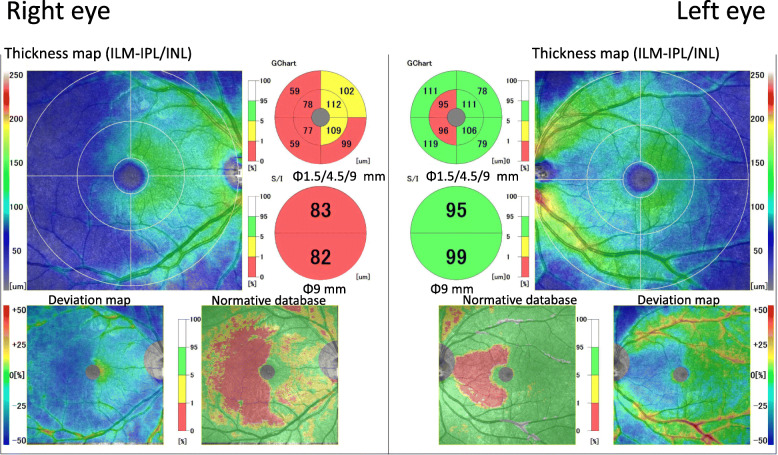
Macular cube scan images with spectral domain optical coherence tomography (SD-OCT). Fourteen months after the accident, the thickness maps show a decrease in the ganglion cell complex (GCC) thickness in left hemilateral sector of both eyes. The hemilateral pattern of retinal ganglion cell (RGC) loss can be clearly seen in the color-coded GCC deviation map

**Progress:** Follow-up examination showed that the misidentification of blue as green in the left hemilateral field had improved approximately 1 week after the injury. Three months after the injury, the patient reported that colors in left hemilateral fields were slightly desaturated compared to the normal side but it was almost unnoticeable in daily life. In addition, the awareness of left hemilateral field dyschromatopsia disappeared completely 8 months after the injury. Although, the sensitivity in the left hemilateral field of both eyes was improved in the SWAP tests, a slight decrease was still present 22 months after the injury (Fig. 2c). OCT showed a thinning of the GCC in the area corresponding to the left hemilateral dyschromatopsia at 14 months after the trauma in both eyes (Fig. 4). At 20 months, no progress of GCC thinning was observed. Throughout the course, there were no accompanying symptoms.

## Discussion and conclusions

Acquired cerebral color anomalies are classified as achromatopsias or dyschromatopsias. They can be associated with a neoplasm, a vascular accident, a trauma and encephalitis [8–10]. In typical cases of achromatopsia, there are a variety of complaints such as being aware of the decrease in both lightness and saturation for all colored objects, and they appear gray. Zeki and colleagues reported complete color blindness in cases of cerebral achromatopsia. During the recovery phase, red is the color usually identified first, and the blue-green system is last to recover [11–14]. The achromatopsia does not improve in some patients, and there are cases that can improve to dyschromatopsia depending on the degree or site of the cerebral injury.

Our case was diagnosed with left hemi-dyschromatopsia. In cerebral color anomalies, the color discrimination ability is reduced especially in differentiating green and blue [3, 11, 12], and the dyschromatopsic deficit is mainly expressed as an accentuation of normal tritatanopic-like tendencies [15]. Our case misidentified blue as green in one-half of the visual field which is also consistent with previous reports [3, 11, 12, 15]. Because of this, no obvious abnormalities could be detected by color vision testing such as panel D-15 and 100-hue tests, which led us to use SWAP in this case. SWAP is a visual field test that uses a blue-on-yellow color increment threshold procedure in which the background light is yellow and the stimulus light is blue. Thus, SWAP is used to assess the functional status of short-wavelength-sensitive (SWS) mechanisms. SWAP was originally developed for the early detection of glaucoma because it can examine alterations of K cells [16]. In addition, it can also detect various blue-yellow abnormalities in eyes with cataracts, retinal color anomalies, and optic neuritis before SAP abnormalities are detected [16, 17].

Our patient had a decrease in sensitivity that was only detected by SWAP testing and not by SAP. So, we believed that the dissociation of SAP and SWAP indicated a disorder of one of the pathways of the SWS mechanism, and we recorded S-cone ERGs to investigate this possibility. The results showed that the S-cone ERGs were normal.

At 14 months after the injury, there was a thinning of the GCC in the OCT images of both eyes in the area corresponding to the left hemilateral sensitivity decrease (Fig. 4). This was considered to be due to the greater susceptibility of K cells and was in agreement with the ERG findings especially the normality of the S-cone ERGs. The GCC thinning occurred by retrograde transsynaptic degeneration as previously reported [18].

The present patient was clearly aware of the symptoms after the cerebral injury, and its onset was distinct and real. Thus, the dyschromatopsia was an acquired color anomaly and not a congenital one. During follow-up, there was an improvement in SWAP which suggests a recovery of the central injury. These findings suggest a plasticity of the cerebral cortex leading to the recovery after an injury.

The site for cerebral color vision abnormalities has been identified in the occipital-temporal lobe [19, 20] and more specifically in the fusiform gyrus and lingual gyrus [2, 21].

Dyschromatopsia occurred in those with acquired lesions of the fusiform gyri, usually bilaterally but sometimes unilaterally [15, 19, 22]. Bouvier reported that color perception may involve many cortical processing sites rather than a single region [19]. More recently, it has been reported that multiple regions with color-related activity, including the more anterior cortex, are the responsible sites [21, 23]. A complex network of processing streams in the occipitotemporal cortex is essential to several aspects of color-related cognition [24].

In our case, possibly there was mild damage in the anterior section of fusiform gyrus due to the brain contusion in the right anterior temporal lobe and it caused color anomaly in the left hemifield. The cerebral dyschromatopsia was caused by a head injury which is rare because the patient had only a color vision abnormality in the left hemilateral field with no other symptoms such as facial agnosia, geographical disorientation, visual field defects, and obvious abnormalities of color tests. Our results indicate that local injury to the anterior section of the fusiform gyrus can cause mild cerebral dyschromatopsia with no other co-existing conditions.

In conclusion, our results showed that even though the subjective symptoms resolved early, a reduced sensitivity of SWAP remained, and optical coherence tomography (OCT) showed retrograde GCC thinning at 14 months after the brain injury. This indicates that even if MRI shows only subtle damage of the brain, it is important to listen to subjective symptoms in detail and to do various tests including SWAP and OCT at the early stage. Cerebral dyschromatopsia which changes over time and possibly disappears could be one of important signs for the presence of subtle brain damage.

## Data Availability

The data and material of the current study are available from the corresponding author on a reasonable request.

## Abbreviations

ERG: Electroretinography
GCC: Ganglion cell complex
MRI: Magnetic resonance imaging
OCT: Optical coherence tomography
SWAP: Short-wavelength automated perimetry
SWS: Short-wavelength-sensitive
SAP: Standard automated perimetry
